# A systematic review on the use of the breast lesion excision system in breast disease

**DOI:** 10.1186/s13244-019-0737-3

**Published:** 2019-05-02

**Authors:** Wendelien B. G. Sanderink, Babette I. Laarhuis, Luc J. A. Strobbe, Ioannis Sechopoulos, Peter Bult, Nico Karssemeijer, Ritse M. Mann

**Affiliations:** 1 Department of Radiology and Nuclear Medicine, Radboud University Medical Center, Nijmegen, the Netherlands; 2 Department of Surgical Oncology, Canisius Wilhelmina Hospital, Nijmegen, the Netherlands; 3 Department of Pathology, Radboud University Medical Center, Nijmegen, the Netherlands

**Keywords:** Breast, Biopsy, Vacuum, Breast cancer, Minimally invasive surgical procedures

## Abstract

**Purpose:**

To outline the current status of and provide insight into possible future research on the breast lesion excision system (BLES) as a diagnostic and therapeutic device.

**Methods:**

A systematic search of the literature was performed using PubMed, Embase, and the Cochrane databases to identify relevant studies published between January 2002 and April 2018. Studies were considered eligible for inclusion if they evaluated the diagnostic or therapeutic accuracy or safety of BLES.

**Results:**

Ultimately, 17 articles were included. The reported underestimation rates of atypical ductal hyperplasia and ductal carcinoma in situ (DCIS) ranged from 0 to 14.3% and from 0 to 22.2%, respectively. Complete excision rates for invasive ductal carcinoma and DCIS ranged from 5.3 to 76.3%. Bleeding was the most frequently reported complication (0–11.8%). Device-related complications may arise, with an empty basket being the most common (0.6–3.6%). Thermal damage of the specimen, caused by the use of a radiofrequency cutting wire, was reported in eight of the included studies. Most thermal artifacts were reported as superficial and small (0.1–1.9 mm).

**Conclusions:**

The BLES, an automated, image-guided, single-pass biopsy system for breast lesions using radiofrequency is designed to excise and retrieve an intact tissue specimen. It is an efficient and safe breast biopsy method with acceptable complication rates, which may be used as an alternative to vacuum-assisted biopsies. The variable rate of complete excision raises questions about the possibility to use BLES as a therapeutic device for the excision of small lesions. Further research should focus on this aspect of BLES.

## Key points

- The Breast Lesion Excision System is designed to excise and retrieve a single intact tissue specimen.
- Reported underestimation rates of atypical ductal hyperplasia (ADH) and ductal carcinoma in situ (DCIS) ranged from 0 to 14.3% and from 0 to 22.2%, respectively.
- Complete excision rates for IDC and DCIS ranged from 5.3 to 76.3%.
- Complications are infrequent and comparable with vacuum-assisted biopsy (VAB).

## Introduction

Breast cancer is the most frequently diagnosed cancer and one of the leading causes of cancer death in women worldwide [1]. The prevalence and incidence of breast cancer have increased over the last 25 years in most countries. Due to increased awareness and screening, up to 53% of cancers are smaller than 2 cm and asymptomatic at detection [2, 3]. Therefore, technologies aimed at achieving minimally invasive complete resection are being investigated.

**Fig. 1:**
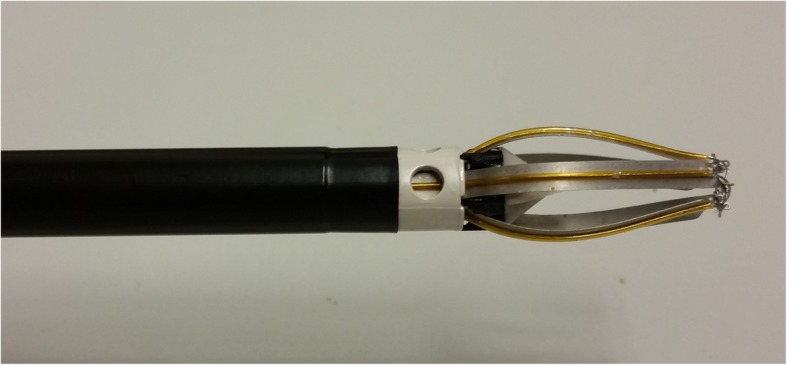
BLES probe

**Fig. 2:**
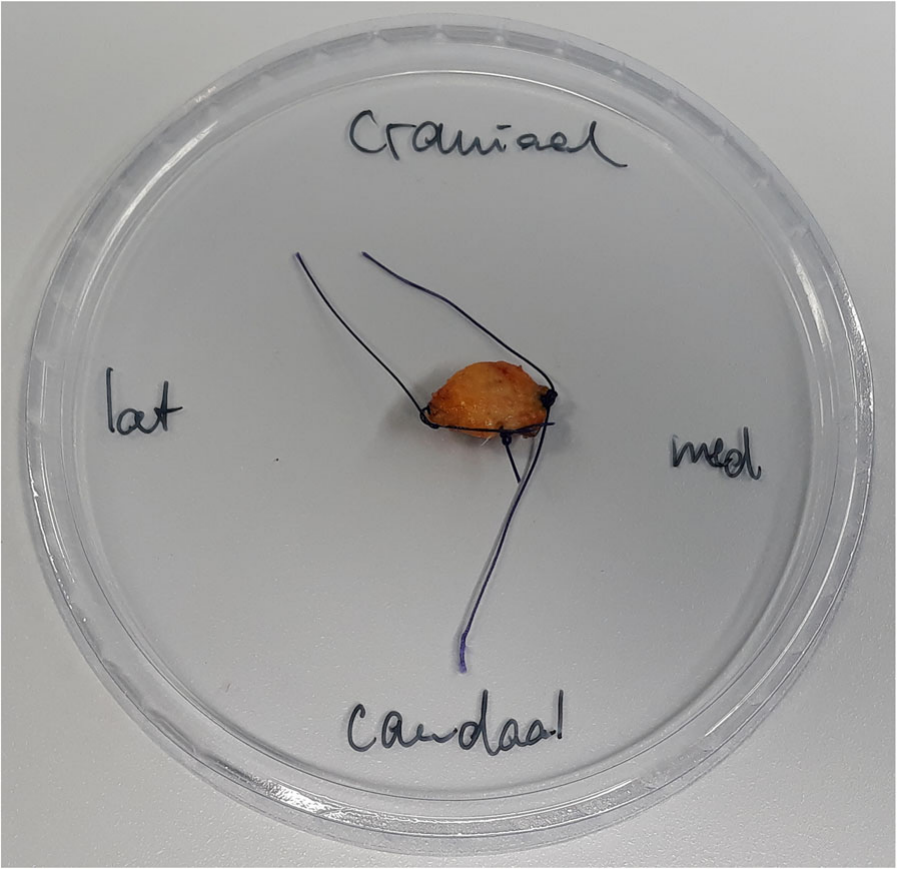
Specimen obtained with the BLES probe

Recently, the breast lesion excision system (BLES) has been developed, which is an automated, image-guided, single-pass biopsy system using radiofrequency (RF). This device is designed to extract entire breast lesions, keeping the tissue architecture intact. The device consists of a probe that can be inserted through a small skin incision of 6–8 mm, with a sharp blade at the distal end to access the target lesion. Just behind the blade, capture wire electrodes are positioned that, once activated, are pushed forward by a motor in the device handle. In approximately 8 s the device deploys the RF cutting mechanism, enclosing the target lesion. To keep the biopsy cavity clear of fluid, which is essential for RF cutting, vacuum ports are located at the distal end of the probe. The capture snare enclosing the specimen can be retracted after the procedure, and a marker clip can be placed in the biopsy cavity through the biopsy canal. Figures 1 and 2 show the BLES probe and an obtained specimen, respectively.

As opposed to other breast biopsy devices, the aim of BLES is to excise and retrieve an intact breast tissue specimen, rather than to obtain fragmented samples [4–7], which may not only facilitate easier diagnosis but also might allow for minimally invasive resections.

In this systematic review, we aim to determine the current status of BLES as a potential diagnostic and therapeutic device in patients with small suspicious or proven (pre-)malignant breast lesions, and its related complications.

## Methods

### Search strategy

A search of the literature was performed in order to identify all articles that examined the diagnostic accuracy, therapeutic efficiency, related complications, and/or thermal damage of BLES in patients with suspicious breast lesions. We searched for articles in PubMed, Embase, and the Cochrane database to identify English language, peer-reviewed articles published between January 1, 2002 and April 24, 2018. The search terms included: breast, percutaneous, intact, specimen, sample, biopsy, breast lesion, excision and radiofrequency, in various combinations. A full list of all performed searches is given in Table 5 in the Appendix. Furthermore, the reference lists of all included articles were manually searched for relevant references.

### Study selection

The search in PubMed and Embase generated 531 and 261 articles, respectively. The Cochrane Library was manually searched, yielding no relevant articles. Duplicate articles were manually filtered using the bibliographic EndNote database, version X8 (Thomas Reuters, New York City, NY, USA), and 537 potentially relevant articles remained.

Titles and abstracts of the remaining articles were evaluated by two authors (WS and BL). Articles were included only if they met all of the following criteria: (I) BLES or a prototype was used as a diagnostic or therapeutic device; (II) a minimum sample size of 10 patients with suspicious lesions referred for breast biopsy was included; (III) stereotactic or ultrasound guidance was used; (IV) the BLES procedure was followed by open surgery in malignant cases, or clinical follow-up of at least 1 year if surgery was not indicated.

### Data extraction, statistics, and quality assessment

The following characteristics were, if available, collected: first author, publication year, country, study design, study period, number of patients, mean age, number of lesions, type of lesions, lesion size, guidance modality, used needle size, procedural success rate, histological data, underestimation rates, complete excision rate, frequency and type of complications, thermal artifacts, and procedural problems. There was no agreement between the papers about the definition of complete excision. Therefore, these definitions were also collected. Results are presented as aggregated data from individual studies.

Underestimation rates for invasive and in situ malignant disease associated with the detection of atypical ductal hyperplasia (ADH) and ductal carcinoma in situ (DCIS) in the biopsy specimens were used to determine the diagnostic accuracy of the BLES. “ADH underestimation” was defined as the percentage of ADH lesions on BLES specimen upgraded to DCIS or invasive cancer at subsequent excision. DCIS underestimation was defined as the percentage of DCIS lesions on BLES biopsy upgraded to invasive cancer in the surgical specimen. Complete excision rate was defined as the fraction of BLES excisions with ADH, DCIS, or invasive cancer that were negative at subsequent surgical excision (i.e., no residual lesion was found).

The quality of the included studies was evaluated by the same two independent observers using the Quality Assessment of Diagnostic Accuracy Studies 2 (QUADAS-2) scoring system [8]. This checklist comprises four domains: patient selection, index test, reference standard, and flow and timing. Not all signaling questions were relevant to assess the study quality for the present review. Two signaling questions were added to the QUADAS-2 scoring system: at the index test domain, the signaling question: “Physicians who performed the index test had appropriate training or the first patients were excluded to account for a learning curve” and at the reference standard domain: “Were patients who did not receive the reference standard specified?” Table 6 in the Appendix shows how the QUADAS-2 score was adapted for this review. Inequalities in scoring by the observers were subsequently resolved by consensus.

Meta-analysis was not performed due to heterogeneity across studies regarding patient selection, definition of success criteria, and presence or absence of surgical verification of results.

## Results

### Studies

**Fig. 3:**
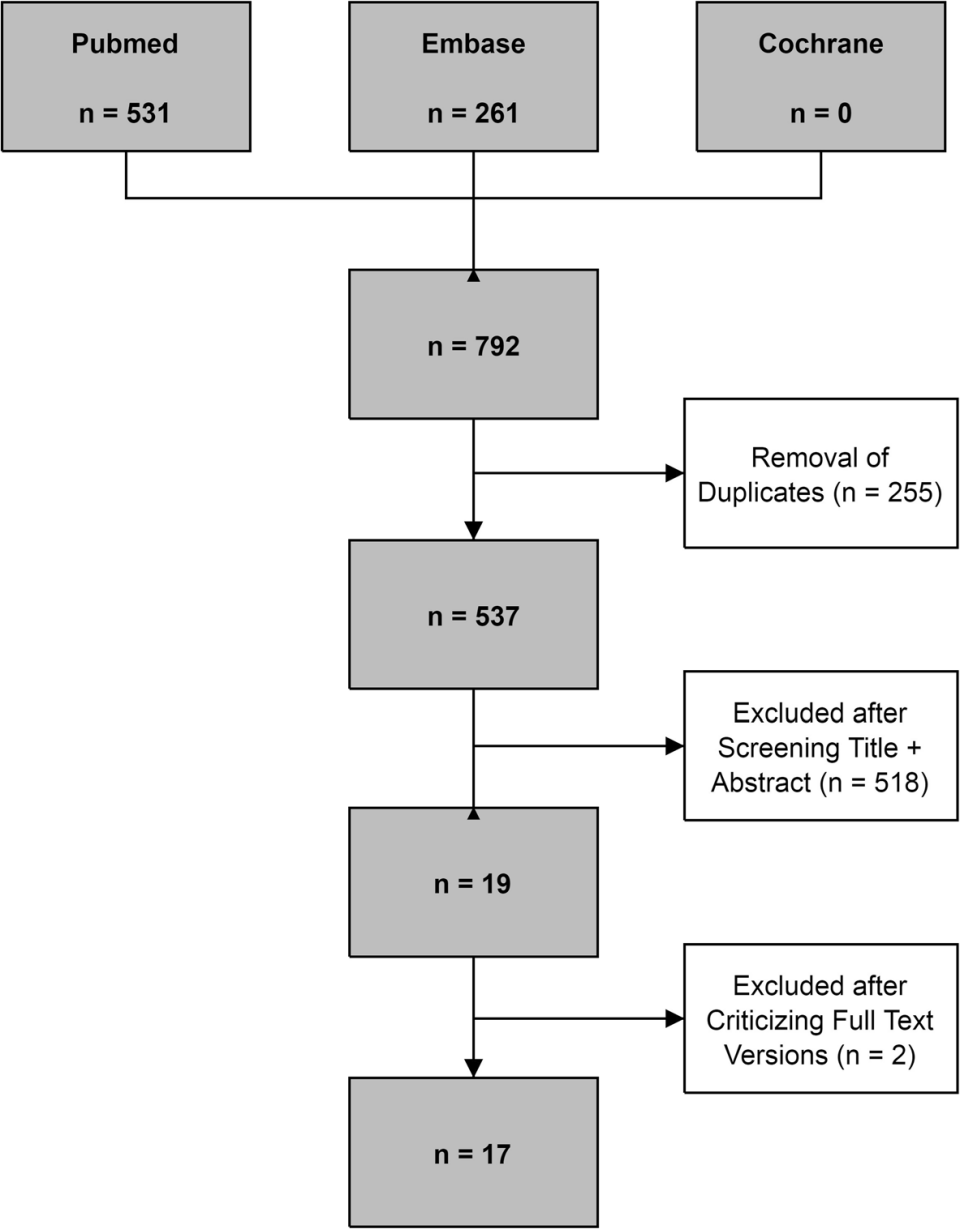
Flowchart of systematic review

Five hundred thirty-seven potential relevant articles remained after the search. Five hundred eighteen articles were excluded because they did not use the BLES device or a prototype. We identified 19 full-text versions of studies that used the BLES as a diagnostic or therapeutic device and that fulfilled all the inclusion criteria [4–7, 9–23]. Figure 3 shows the results of the study search and identification of eligible studies. We did not retrieve any additional items after reference screening. The study by Fine et al. [19] was excluded because although a comparable device was used, it was not a prototype of BLES. Citgez et al. [18] published their findings as an abstract only and was therefore excluded.

The characteristics of the 17 included studies are shown in Table 1. All studies were observational: 10 studies enrolled participants prospectively while 7 studies were retrospective. In all of these studies, included patients had known mammographic abnormalities (masses or suspicious calcifications) with a mean lesion size on imaging ranging from 5.7 mm to 12.7 mm (reported range, 1 mm − 76 mm). Mean age ranged from 50.5 years to 61.8 years (reported range, 21 years–93 years). The number of lesions ranged from 19 to 1170. Ten studies used stereotactic guidance during the BLES procedure [4, 6, 7, 9, 11, 13, 15, 16, 20, 22]. Six studies used stereotactic or ultrasound guidance [5, 10, 12, 14, 21, 23]; only Graham [17] performed all BLES procedures with ultrasound guidance.

**Table 1:** Characteristics of the included studies

Authors, publication year and country	Study type; Study period	Number of lesions^a^	Entry criteria	Age (years)^b^	Basket size (mm)	Lesion size (mm)^b^	Guidance type
Sie et al. 2006, USA (4)	Retrospective; 2002–2004	742	Breast lesions with microcalcifications	NA	10, 15	NA	Stereotactic
Killebrew et al. 2006, USA (9)	Retrospective; 2003–2004	800	Mammographic lesions presenting as microcalcifications	NA	10, 15	NA	Stereotactic
Allen et al. 2011, UK (5)	Prospective; 2007-NA	76	Sub-centimeter breast lesions	NA	15, 20	7.1 (2–10)	Ultrasound + stereotactic
Seror et al. 2011, France (10)	Prospective; 2008–2009	166	Microcalcifications	55.7 (31–93)	12, 15, 20	8.1 (2–25)	Ultrasound + stereotactic
Diepstraten et al. 2011, Netherlands (11)	Prospective; 2010	19	Mammographic lesions presenting as microcalcifications	59 (37–74)	15, 20	8 (2–76)	Stereotactic
Whitworth et al. 2011, USA (6)	Prospective; 2006–2010	1170	Mammographic lesion recommended for image-guided core-needle breast biopsy	NA	15, 20	NA	Stereotactic
Razek et al. 2013, Egypt (12)	Prospective; 2012	80	Small breast lesions with unclassified microcalcifications	NA (21–55)	15, 20	9 (4–16)	Ultrasound + stereotactic
Medjhoul et al. 2013, France (13)	Retrospective; 2010–2012	31	Mammographic lesions presenting as calcifications or masses	61.2 (NA)	12, 15, 20	10 (3–38)	Stereotactic
Al-Harethee et al. 2013, Greece (7)	Prospective; 2008–2010	134	Mammographic lesions presenting as microcalcifications, solid lesions or asymmetric density	51.3 ± 10.3	12, 15, 20	NA	Stereotactic
Allen et al. 2014, UK (14)	Prospective; 2007–2009	41	Sub-centimeter breast lesions	NA	15, 20	5.7 (1–10)	Ultrasound + stereotactic
Al-Harethee et al. 2015, Greece (15)	Prospective; 2009–2012	273	Suspicious, non-palpable mammographic lesions	54.4 ± 10.4	12, 15, 20	NA	Stereotactic
Scaperrotta et al. 2016, Italy (16)	Retrospective; 2010–2014	105	Mammographic lesions presenting as microcalcifications measuring up to 1 cm	55 (38–81)	15, 20	≤ 10 (NA)	Stereotactic
Graham, 2017, USA (17)	Prospective; 2007–2014	461	US visualized lesions measuring up to 2 cm	NA (23–88)	12, 15, 20	< 20 (NA)	Ultrasound
Milos et al. 2017, Austria (20)	Retrospective; 2011–2015	34	Microcalcifications	55 (31–75)	12, 15, 20	8 (4–15)	Stereotactic
Sklair-Levy et al. 2017, Israel (21)	Prospective; 2012–2016	111	Benign or atypical high-risk lesions	50.5 (21–91)	12, 15, 20	< 20 (NA)	Ultrasound + stereotactic
Papapanagiotou et al. 2017, Greece (22)	Retrospective; 2010–2014	50	Pathological diagnosis of a carcinoma lesion	61.8 (43–80)	12, 15, 20	12.7 (1.5–30)	Stereotactic
Niinikoski et al. 2018, Finland (23)	Retrospective; 2011–2016	80	Histological or cytological and radiological suspicion of an intraductal papilloma	60 (25–84)	12, 15, 20	7 (3–16)	Ultrasound + stereotactic

*NA* not available

^a^Number of lesions treated with BLES procedure

^b^Unless otherwise stated, data are means, with ranges in parentheses

Overall, 4373 BLES biopsies were performed in 17 studies. The procedure was technically successful in 4320 procedures, with success rates varying between 84% and 100%. Eight studies were performed for diagnostic purposes only [4, 6, 7, 9, 11, 13, 15, 17]. In two studies, one or more biopsies were performed to remove benign lesions for which histology was already known [21, 23]. One study aimed at a complete, tumor-free margin excision of small solid carcinomas [22].

### Quality assessment

**Fig. 4:**
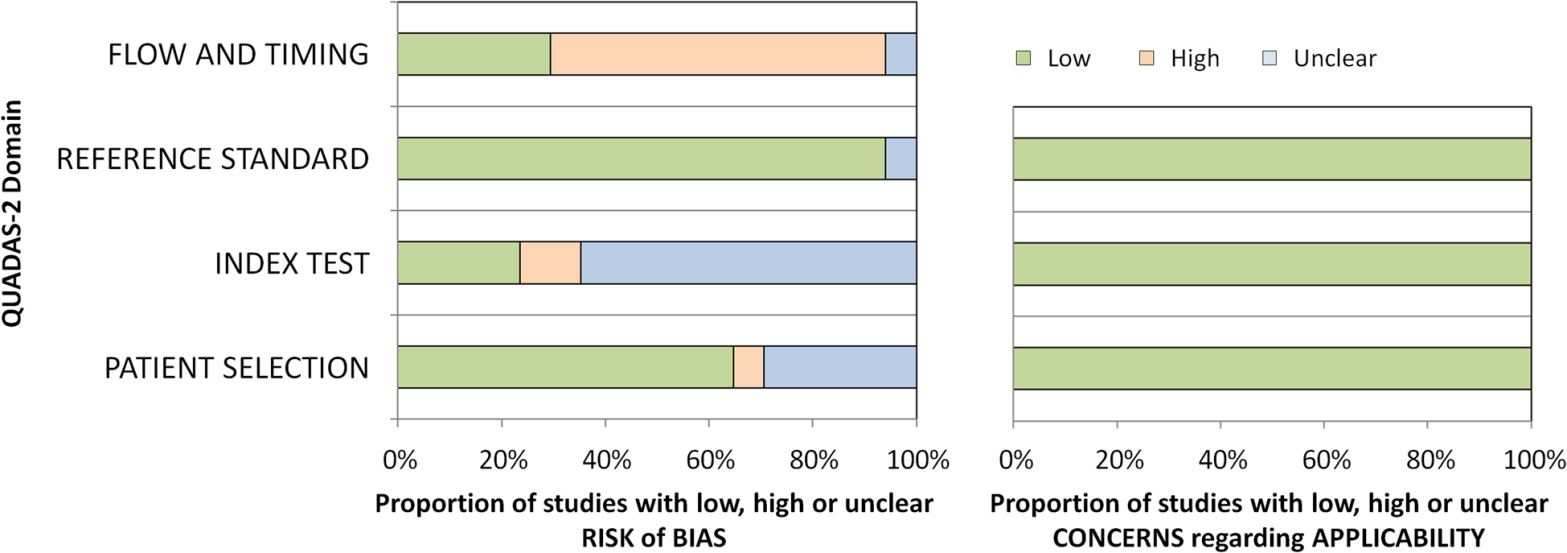
QUADAS-2 graph demonstrates the risk of bias and the applicability of assessment results

The results of bias risk and applicability according to the QUADAS-2 evaluation are summarized in Fig. 4. In five studies, the risk of bias in patient selection was considered uncertain due to unreported details [5, 11–14]. The study of Scaperrotta et al. [16] was considered to present a high risk of bias in patient selection since patients were not consecutively enrolled. The decision to use the BLES was entirely based on the radiologist’s assessment. Presence of bias risk of the index test was uncertain in 11 studies [6, 7, 9, 12, 13, 15–17, 20–22] and high in two studies [10, 23]. The risk of bias in the “reference standard” domain was generally scored low. Only the study by Al-Harethee et al. [7] had an unclear bias risk because there was no information available about the used reference standard. Admittedly, this was beyond the scope of their study. The risk of bias in the flow and timing was generally scored as high, because not all patients with a high-risk lesion (HRL) or malignancy based on the BLES received surgical excision. Only five studies [15, 20–23] were scored with a low risk of this bias and one [7] with an unclear risk of bias. All studies were deemed applicable to the research question. In short, no studies were excluded based on the quality assessment.

### Diagnostic accuracy

BLES was, in most studies, used as a diagnostic tool for breast abnormalities for which histopathology was not yet available. Diagnostic accuracy was usually reported as the underestimation rates of more aggressive disease in lesions diagnosed as ADH and/or DCIS by BLES. The ADH and DCIS underestimation rates ranged from 0 to 14.3% and from 0 to 22.2%, respectively. In addition, the diagnosis was upgraded by subsequent BLES excision after ADH, DCIS, or intraductal papilloma was found on core needle biopsy (CNB) or fine needle aspiration (FNA) cytology in 2.7%, 0.9%, and 19.7% of cases, respectively [21, 23]. Results of all individual studies are shown in Table 2.

**Table 2:** Summary of underestimation rates of BLES in literature

First author	ADH underestimation rate n/N (%)	DCIS underestimation rate n/N (%)
Sie et al. (4)	3/32 (9.4)	6/115 (5.2)
Killebrew et al. (9)	NA	1/31 (3.2)
Allen et al. (5)	NA	NA
Seror et al. (10)	0/4 (0)	6/27 (22.2)
Diepstraten et al. (11)	NA	0/3 (0)
Whitworth et al. (6)	3/32 (9.4)^a^	NA
Razek et al. (12)	0/14 (0)	0/8 (0)
Medjhoul et al. (13)	0/4 (0)^b^	1/9 (11.1)
Al-Harethee et al. (7)	NA	NA
Allen et al. (14)	0/6 (0)	0/11 (0)
Al-Harethee et al. (15)	NA	NA
Scaperrotta et al. (16)	NA	5/32 (15.6)
Graham (17)	1/38 (2.6)^b^	0/8 (0)
Milos et al. (20)	2/14 (14.3)^b^	0/10 (0)
Sklair-Levy et al. (21)	NA	NA
Papapanagiotou et al. (22)	NA	0/5 (0)
Niinikoski et al. (23)	NA	NA

*ADH* atypical ductal hyperplasia; *DCIS* ductal carcinoma in situ; *n* number of DCIS/IC reference (initially ADH) or number of IC reference (initially DCIS); *N* total number of initially ADH or DCIS; *NA* not available

^a^23/32 based on open surgical excision, 6/32 based on image follow-up

^b^High-risk lesion underestimation

### Therapeutic accuracy

Only two studies have investigated the ability to use BLES as a therapeutic device for removal of lesions for which histological results were already obtained by other means [21, 23]. An additional three studies assessed the therapeutic value of the system without prior knowledge of histology [16, 20, 22], and several studies reported the complete excision rate of biopsied lesions, even though they did not aim to excise the entire lesion [4, 5, 9–14]. See Table 3.

**Table 3:** Reported complete excision rates of benign, ADH, DCIS, and IDC lesions

First author	Definition complete excision	Benign complete excision rate n/N (%)	ADH complete excision rate n/N (%)	DCIS complete excision rate n/N (%)	IDC complete excision rate n/N (%)	Total complete excision rate n/N (%)
Sie et al. (4)	No residual disease at surgical specimen	NA	21/32 (65.6)	1/115 (0.9)	NA	22/147 (15)
Killebrew et al. (9)	BLES produced a diagnosis of DCIS or ADH and surgical biopsy resulted in a benign diagnosis with no evidence of IDC, DCIS, or ADH	NA	NA	12/31 (38.7)	NA	12/31 (38.7)
Allen et al. (5)	No residual disease at surgical specimen	5/6 (83.3)	NA	4/6 (66.7)^a^	3/6 (50)^b^	12/18 (66.7)
Seror et al. (10)	No residual disease at surgical specimen	0/1 (0)	2/9 (22.2)^c^	11/27 (40.7)	1/7 (14.3)^d^ 0/2 (0)^e^	14/46 (30.4)
Diepstraten et al. (11)	NA	NA	NA	1/19 (5.3)^f^	NA	1/19 (5.3)
Whitworth et al. (6)	NA	NA	NA	NA	NA	NA
Razek et al. (12)	1 mm free margin after BLES	NA	20/24 (83.3)^g^	6/8 (75)	3/6 (50)	29/38 (76.3)
Medjhoul et al. (13)	No residual disease at surgical specimen	NA	3/4 (75)	NA	0/10 (0)	3/14 (21.4)
Al-Harethee et al. (7)	NA	NA	NA	NA	NA	NA
Allen et al. (14)	No residual disease at surgical specimen	NA	3/6 (50)	6/11 (54.5)	5/8 (62.5)	14/25 (56)
Al-Harethee et al. (15)	NA	NA	NA	NA	NA	NA
Scaperrotta et al. (16)	No residual disease at surgical specimen	NA	NA	16/32 (50)	1/3 (33.3)^d^ 2/4 (50)^h^	19/39 (48.7)
Graham (17)	NA	NA	NA	NA	NA	NA
Milos et al. (20)	BLES produced a HRL or malignant lesion and the surgical specimen did not contain any remnants of the initial malignant or high-risk lesion	NA	8/14 (57.1)^i^	3/10 (30)	4/10 (40)	15/34 (44.1)
Sklair-Levy et al. (21)	NA	NA	NA	NA	NA	NA
Papapanagiotou et al. (22)	No residual disease at surgical specimen	NA	NA	4/5 (80)	21/45 (46.7)	25/50 (50)
Niinikoski et al. (23)	Complete excision was based on histological margin status of the BLES sample	19/43 (44.2)^j^	6/10 (60)^i^	2/3 (66.7)	0/2 (0)	27/58 (46.6)

Values as presented in the articles; *ADH* atypical ductal hyperplasia*; BLES* breast lesion excision system; *DICS* ductal carcinoma in situ; *HRL* high-risk lesions; *IDC* invasive ductal carcinoma; *n* number of (benign/ADH/DCIS or IDC) complete excisions; *N* total number of initially benign/ADH/DCIS or IDC; *NA* not available;

^a^Low-grade DCIS

^b^IDC and other malignant lesions

^c^Lesions with cell atypia

^d^IDC complete excision rate

^e^Invasive lobular carcinoma (ILC) complete excision rate

^f^Based on post-biopsy mammographic follow-up and specimen radiography

^g^ADH, atypical lobular hyperplasia (ALH) and lobular carcinoma in situ (LCIS)

^h^DCIS with micro-invasion complete excision rate

^i^High-risk lesion complete excision rate

^j^Benign intraductal papilloma

The median of all reported DCIS complete excision rates was 50% (range, 0.9–80%) [4, 5, 9–12, 14, 16, 20, 22, 23]. For complete excision rates of ADH, studies reported a median of 60% (range, 22.2–83.3%) [4, 10, 12, 13, 20, 23]. For complete excision rates of invasive ductal carcinoma (IDC), the median was 43% (range, 0–62.5%) [5, 10, 12–14, 16, 20, 22, 23].

### Complications and procedural problems

Studies reported various complications associated with the use of BLES (Table 4), although complications were infrequent and usually mild. The most common complications were bleeding (0–11.8%), hematoma (0–8.8%), infection (0–5.3%), wound leakage (5.3%), wound healing problems (0.9–5.2%), and skin burn (0–1.5%) [4, 5, 7, 9–14, 17, 20–23]. In addition to patient-related complications, device- and procedure-related problems were also reported: wire break (0.6–1%), basket failed to deploy (0.7–2%), incorrect guidance (0.9–1.2%), and an empty basket after the procedure (0.6–3.6%) [7, 9, 10, 15, 16, 21, 22]. In the case of device-related complications that lead to an unsuccessful procedure, the use of a second probe was necessary to complete the procedure.

**Table 4:** Reported patient-related and device-related complications

First author	Infection *n* (%)	Bleeding *n* (%)	Hematoma *n* (%)	Wound leakage *n* (%)	Wound healing problems *n* (%)	Skin burning *n* (%)	Empty basket *n* (%)	Wire break *n* (%)	Incorrect guidance *n* (%)	Basket failed to deploy *n* (%)	Thermal artifact mm
Sie et al. (4)	1 (0.1)	0 (0)	0 (0)	NA	NA	0 (0)	NA	NA	NA	NA	0.1–1
Killebrew et al. (9)	NA	NA	0 (0)	NA	NA	NA	29 (3.6)	8 (1.0)	NA	NA	0.2–1
Allen et al. (5)	0 (0)	NA	1 (1.3)	NA	NA	NA	NA	NA	NA	NA	< 1
Seror et al. (10)	NA	NA	1 (0.6)	NA	NA	NA	1 (0.6)	1 (0.6)	2 (1.2)	NA	0.2
Diepstraten et al. (11)	1 (5.3)	1 (5.3)	NA	1 (5.3)	NA	NA	NA	NA	NA	NA	0.4–1.9
Whitworth et al. (6)	NA	NA	NA	NA	NA	NA	NA	NA	NA	NA	NA
Razek et al. (12)	NA	NA	3 (3.8)	NA	NA	NA	NA	NA	NA	NA	< 1
Medjhoul et al. (13)	0 (0)	NA	1 (3.1)	NA	NA	NA	NA	NA	NA	NA	NA
Al-Harethee et al. (7)	4 (3.0)	9 (6.7)	5 (3.7)	NA	7 (5.2)	2 (1.5)	NA	NA	NA	1 (0.7)	NA
Allen et al. (14)	0 (0)	NA	1 (2.4)	NA	NA	NA	NA	NA	NA	NA	< 1
Al-Harethee et al. (15)	NA	NA	NA	NA	NA	NA	NA	NA	NA	3 (1.1)	1–2
Scaperrotta et al. (16)	NA	NA	NA	NA	NA	NA	1 (1.0)	NA	NA	NA	NA
Graham (17)	1 (0.3)	NA	27 (6.7)	NA	4 (0.9)	2 (0.5)	NA	NA	NA	NA	NA
Milos et al. (20)	0 (0)	4 (11.8)	3 (8.8)	NA	NA	NA	NA	NA	NA	NA	NA
Sklair-Levy et al. (21)	NA	NA	4 (3.6)	NA	NA	NA	NA	NA	1 (0.9)	NA	NA
Papapanagiotou et al. (22)	1 (2)	NA	1 (2)	NA	NA	NA	NA	NA	NA	1 (2)	NA
Niinikoski et al. (23)	NA	NA	1 (1.25)	NA	NA	1 (1.25)	NA	NA	NA	NA	NA

*NA* not available; *n* number of reported complications;

Thermal damage to the specimen is regularly present due to the use of the RF-based cutting wire and reported by several studies that evaluated the BLES. However, the reported thermal artifacts were mostly superficial and small. The affected tissue thickness ranged overall from 0.1 mm to 1.9 mm [4, 5, 9–12, 14, 15] and was more extensive toward the pole of the ellipsoid specimen (0.7–1.9 mm) than centrally (0.1–1 mm).

## Discussion

This systematic review reports on 17 studies on the diagnostic and therapeutic accuracy, and complications of BLES in patients with suspicious breast lesions. A (pooled) meta-analysis was not performed because of heterogeneity in study design and included patient populations. Overall, the procedural success rates are high. Despite the fact that most studies did not aim to remove lesions entirely, complete excision occurs regularly, depending on the type of lesion. Finally, complications are infrequent and usually mild. Although technical failures might occur due to specific properties of the BLES, they are infrequent. Although the device is only approved for diagnostic purposes it certainly has the potential to be used as a therapeutic device.

The overall study quality of all included studies is reasonably high according to the QUADAS-2 score. According to the instructions for use, the QUADAS-2 tool was tailored for this systematic review, which means that some signaling questions were added or omitted, as provided in Table 6 in the Appendix. The signaling question “Physicians who performed the index test had appropriate training or the first patients were excluded for the learning curve?” was added because the likely present learning curve may have an influence on other variables, such as success rate, complications, and technical failure. Also, the letter of Michalopoulos et al. describes that it has been estimated that for dedicated breast radiologists approximately four procedures and for those without previous VAB experience nine procedures are required to gain experience with the BLES technique [24]. The signaling question “Were patients who did not receive the reference standard specified?” was added because surgical excision is mandatory for patients with a malignant or HRL in the index test. Underestimation rates of biopsies containing ADH and DCIS are commonly used to determine the accuracy of percutaneous biopsy techniques [25, 26]. Multiple studies focused on ADH or DCIS underestimation rates of VAB and of CNB with varying needle sizes. In a systematic review of VAB, Yu et al. [27] reported a pooled ADH and DCIS underestimation of 20.9% (95% CI 17.7–24.5%) and 11.2% (95% CI 9.8–12.8%), respectively. Reported underestimation rates for CNB are generally higher: 44.2% (95% CI 36.0–52.5%) and 22.8% (95% CI 19.0–26.5%), respectively [28]. In the current review of BLES, ADH and DCIS underestimation rates are therefore in the same order of magnitude as those reported for VAB. The en bloc resection obtained with BLES preserves lesion architecture, which may make subsequent histopathological classification easier, facilitating discrimination between atypical and (pre-)malignant lesions. Furthermore, the possibility to examine the margins of the lesion allows determination of the excision completeness, which is crucial for high-risk or (pre-)malignant lesions [29]. However, in normal clinical situations, the BLES will not be the first choice biopsy device, because it is more invasive, expensive, and requires adequate training.

The varying rates of complete excision suggest that future research should focus on the characteristics of lesions for which BLES can be used for therapeutic resection. It should be noted that complete excision rates of clusters of suspicious microcalcifications under stereotactic guidance are low. The cluster size of microcalcifications on mammography is anyhow poorly correlated with pathological tumor size in both DCIS and invasive disease [30]. Therefore, it is highly recommended to focus future research on lesions that are clearly visible on mammography or US. It would be appropriate to modify the needles to make them appropriate for MRI-guided biopsy (ferromagnetic-material-free) so that the lesion size could be measured more precisely and needle size selection could be adjusted accordingly. It is important to realize that the basket should be large enough to capture the entire lesion when the intended use is therapeutic. An upgraded BLES needle with a diameter of 30 mm is under development. This may further reduce underestimation rates and expand therapeutic possibilities.

Although most studies recorded the presence of RF coagulation artifacts, these artifacts are most prominent around the pole of the ellipsoid specimen. A possible explanation is that the precursor electrode is situated at the distal end of the probe and tissue is more exposed to this part. Some studies note that pathologists may have difficulties with interpretation and assessment of edges and margins of lesions obtained with BLES because of these RF artifacts. However, this problem seems to wane when the pathologist gains more experience with BLES samples [9–11, 13, 14]. In fact, most breast pathologists are used to coagulation artifacts at the edges of breast specimens as breast surgeons commonly work with a diathermic knife. Nevertheless, there are some options to minimize the effect of RF artifacts. First, placing local anesthetic fluid effectively around the entire lesion, because dry tissue burns easier. Second, aiming to get the lesion in the middle of the resection specimen, rather than at the distal pole, will reduce the effect of thermal damage to the lesion. Using a larger wand should increase the distance between the RF artifact and the lesion.

The most frequently reported device-related failure was an empty basket. The cause of this failure is unknown but thought to be associated with the presence of excessive (anesthetic) fluid which blocks the RF cutting mechanism or the presence of very fatty breast tissue which melts during the procedure [9, 15, 31]. Unfortunately, in case of an empty cage after biopsy, no salvage technique is available other than marker placement followed by surgical excision, or when the lesion is still visible, an attempt using VAB. In case of a basket deployment failure, a second disposable is necessary, with associated costs, because the system uses single-shot only needles. Adjusting the needles for re-use in the same patient could be a solution.

In conclusion, BLES is a diagnostic device with a diagnostic accuracy at least as good as VAB, as expressed by ADH and DCIS underestimation rates. The technique is safe for use. Disadvantages of BLES are the reported device-specific problems and the fact that only one attempt of lesion removal per needle is possible. Advantages include preservation of lesion architecture, and the possibility to assess lesion margins. Although there is a small risk of thermal damage to the biopsy specimen that might hinder pathological evaluation, this appears limited. BLES therefore offers a viable alternative to VAB. Based upon the balance between advantages and disadvantages, BLES seems most suited for the complete excision of small breast lesions for which a definitive diagnosis is required (e.g., papillomas). Because BLES is minimally invasive and permits margin evaluation, the value of this device may be mainly in the therapeutic field, future research should therefore focus on this. A “treat and resect” study design, in which a BLES excision is immediately followed by a surgical procedure of the biopsy cavity, seems to be most feasible for the evaluation of the potential of the technique for treatment of small cancers. This may depend on the availability of larger basket sizes. It is also important to assess whether it is possible to predict successful tumor extraction based upon patient and tumor characteristics, as adequate patient selection seems mandatory. If such studies are successful, then follow-up studies should be performed in large-scale multi-center settings to evaluate the resection of small invasive carcinomas under local anesthesia only with BLES, followed by additional surgery only if resection margins are positive. Afterward, these patients must be followed for a long period to analyze possible effects on local recurrence and disease-free survival. Also, the improvement, if any, in quality of life should be evaluated. Finally, a cost-effectiveness analysis from a healthcare perspective is necessary to assess the impact on healthcare costs.

## Appendix

**Table 5:** Full list of performed searches

Set	Search statement for PubMed and Embase^a^
#1	Breast lesion excision[tiab]
#2	Breast [tiab] AND Percutaneous excision[tiab]
#3	Breast [tiab] AND Percutaneous biops*[tiab]
#4	Breast [tiab] AND Intact[tiab] AND (Sample*[tiab] OR Specimen*[tiab])
#5	Percutaneous[tiab] AND Biops*[tiab] AND Breast lesion*[tiab]
#6	Biops*[tiab] AND Breast lesion*[tiab] AND Radiofrequency[tiab]
#7	#1 OR #2 OR #3 OR #4 OR #5 OR #6

^a^Limited to English language studies in human females, from January 1, 2002 to April 24, 2018

**Table 6:** Adapted QUADAS-2 score form

Signaling question	Signaling question	Risk of bias	Concerns about applicability
Domain 1: Patient selection			
Was a consecutive or random sample of patients enrolled?	Did the study avoid inappropriate exclusions?	Could the selection of patients have introduced bias?	Are there concerns that the included patients and setting do not match the review question?
Yes: If all consecutive or random samples of subjects were enrolled. No: If subjects were nonrandomly selected. Unclear: If sampling method was unclear.	Yes: If there were no inappropriate exclusion criteria or patients were excluded with appropriate arguments. No: If subjects were excluded based on inappropriate criteria or with inappropriate arguments. Unclear: If selection criteria were unclear	Low risk: If all signaling questions answered ‘yes’. High risk: If ‘no’ was reported for at least one signaling question, or if ‘unclear was reported for more than one signaling question. Unclear risk: If ‘unclear’ was reported for one signaling question.	Low concern: If selected subjects matched the review question and inappropriate exclusions were avoided. High concern: If selected subjects differed from those in the review question. Unclear concern: If there was insufficient information on included subjects and setting.
Domain 2: Index test			
Were the index test results interpreted without knowledge of the results of the reference standard?	Physicians who performed the index test had appropriate training or the first patients were excluded for the learning curve.	Could the conduct or interpretation of the index test have introduced bias?	Are there concerns that the index test, its conduct, or interpretation differ from the review question?
Yes: If the index test results were interpreted without knowledge of the histopathological analysis of surgical specimen or when surgical biopsy only was offered based on index test results. No: If the index test results were interpreted with knowledge of the histopathological analysis of surgical specimen. Unclear: If it was unclear whether index test results were interpreted independently of the histopathological analysis of surgical specimen.	Yes: If an appropriate training for the physicians was defined or the first patients were excluded for the learning curve. No: If physicians had no appropriate training or no patients were excluded for the learning curve. Unclear: If it was unclear whether the physicians had an appropriate training or patients were excluded for the learning curve.	Low risk: If all signaling questions answered ‘yes’. High risk: If ‘no’ was reported for at least one signaling question, or if ‘unclear was reported for more than one signaling question. Unclear risk: If ‘unclear’ was reported for one signaling question.	Low concern: If the index test was performed as described in the review question. High concern: If the index test differed from those specified in the review question. Unclear concern: If there was insufficient information available
Domain 3: Reference standard			
Is the reference standard likely to correctly classify the target condition?	Were patients who did not receive the reference standard specified?	Could the reference standard, its conduct, or its interpretation have introduced bias?	Are there concerns that the target condition as defined by the reference standard does not match the review question?
Yes: All patients received histopathological analysis of surgical specimen. No: Some or all patients received any other reference standard or no reference standard. Unclear: Reference standard is not stated.	Yes: If the reference standard was recommended for all malignant lesions and High Risk lesions, but was refrained with appropriate arguments. No: If the reference standard was not offered or without appropriate arguments. Unclear: Exclusions were not stated.	Low risk: If all signaling questions answered ‘yes’. High risk: If ‘no’ was reported for at least one signaling question, or if ‘unclear was reported for more than one signaling question. Unclear risk: If ‘unclear’ was reported for one signaling question.	Low concern: If pathological analysis of surgical specimen or mammographic follow-up was used. High concern: If pathological analysis of surgical specimen or mammographic follow-up was not used. Unclear concern: If insufficient information was provided in the report.
Domain 4: Flow and timing			
Did all patients receive the reference standard?	Were all patients included in the analysis?	Could the patient flow have introduced bias?	
Yes: If all eligible subjects with a malignant lesion or high risk lesion received surgical biopsy. No: If not all eligible subjects received the reference standard. Unclear: If this was not clear from the report.	Yes: If all subjects recruited to the study with reference standard results were included in the analysis. No: If not all recruited subjects with reference standard results were included in the analysis. Unclear: If this was unclear from the report	Low risk: If all signaling questions answered „yes. High risk: If ‘no’ was reported for at least one signaling question, or if ‘unclear was reported for more than one signaling question. Unclear risk: If ‘unclear’ was reported for one signaling question.	

## Abbreviations

ADH: Atypical ductal hyperplasia
BLES: Breast lesion excision system
CNB: Core needle biopsy
DCIS: Ductal carcinoma in situ
FNA: Fine needle aspiration
HRL: High-risk lesion
IDC: Invasive ductal carcinoma
LCIS: Lobular carcinoma in situ
QUADAS-2: Quality Assessment of Diagnostic Accuracy Studies 2
RF: Radiofrequency
VAB: Vacuum-assisted biopsy
